# Enhancing the Clinical Relevance of Al Research for Medication Decision-Making

**DOI:** 10.2196/70657

**Published:** 2025-02-18

**Authors:** Qi Wang, Mingxian Chen

**Affiliations:** 1 Zhejiang Chinese Medical University Hangzhou China; 2 Traditional Chinese Medicine Hospital of Luqiao District Taizhou China; 3 Department of Gastroenterology Tongde Hospital of Zhejiang Province Hangzhou China

**Keywords:** older adults, artificial intelligence, medication, decision-making, data security, patient trust

We are writing to provide comments on the manuscript titled “Investigating Older Adults’ Perceptions of Al Tools for Medication Decisions: Vignette-Based Experimental Survey” by Vordenberg et al [1]. This study addresses a critical area of research, exploring older adults’ trust in artificial intelligence (Al) tools for medication recommendations. While the manuscript presents meaningful insights, several aspects could benefit from further clarification or expansion.

First, older adults may encounter practical challenges when using AI tools, such as difficulties with device operation or complex interfaces. Incorporating user testing or enhanced scenario simulations could optimize the usability of AI tools for older adults. As technology evolves, older adults’ perceptions of AI may change over time or with increased exposure to its use. A comparison of AI recommendations with those of health care professionals may either strengthen trust in AI or create conflicts with medical advice, which could lead to greater tensions in the doctor-patient relationship [2]. Long-term follow-up studies would be valuable in tracking these dynamics.

Second, the study emphasizes the impact of differences among demographic groups but does not explore the cultural and socioeconomic factors that underlie these disparities. The acceptance of AI recommendations by older adults may be influenced by cultural attitudes, psychological factors, income levels, and past medical experiences. These elements may limit the generalizability of the study’s findings across diverse social contexts [3]. Future research should delve deeper into these factors to enhance the applicability of the results.

Third, while the authors have addressed certain issues, we believe that several important considerations remain underexplored. The study assumes controlled scenarios to manage AI-related variables; however, this simplification may be overly idealized and fails to capture the complexity of medication decision-making in real-world contexts [4]. In practice, decisions regarding medication discontinuation are influenced by multiple intersecting factors, including not only the doctor’s advice but also communication and trust between the patient and health care provider, the patient’s financial situation, the need for coordinated care across multiple chronic conditions, and potential side effects following medication cessation. Additionally, the opinions and interventions of family members, as well as the patient’s personal values, may also play significant roles in the decision-making process [5]. Therefore, medication decisions in real-life scenarios are very complex and require a comprehensive consideration of various factors beyond fixed hypothetical scenarios.

Overall, this study makes a significant contribution to understanding older adults’ perceptions of AI in health care. Addressing these limitations could enhance the clinical relevance and robustness of future research. We would like to commend the authors for their efforts and thank the editorial team for providing a platform to publish this important work.

## Abbreviations

AI: artificial intelligence
